# Stimulation of Peanut Seedling Development and Growth by Zero-Valent Iron Nanoparticles at Low Concentrations

**DOI:** 10.1371/journal.pone.0122884

**Published:** 2015-04-22

**Authors:** Xuan Li, Yuechao Yang, Bin Gao, Min Zhang

**Affiliations:** 1 National Engineering Laboratory for Efficient Utilization of Soil & Fertilizer Resources, National Engineering & Technology Research Center for Slow & Controlled Release Fertilizers, College of Resources and Environment, Shandong Agricultural University, Taian, Shandong 271018, China; 2 Department of Soil and Water Science, Tropical Research and Education Center, University of Florida, Homestead, Florida, United States of America; 3 Department of Agricultural and Biological Engineering, University of Florida, Gainesville, Florida, United States of America; NERC Centre for Ecology & Hydrology, UNITED KINGDOM

## Abstract

Because of its strong pollutant degradation ability, nanoscale zerovalent iron (NZVI) has been introduced to soils and groundwater for remediation purposes, but its impacts on plants are still not very clear. In this work, the effects of low concentration (10–320 μmol/L) NZVI particles on seed germination and growth of peanut plants were evaluated. The exposure of peanut seeds to NZVI at all the tested concentrations altered the seed germination activity, especially the development of seedlings. In comparison with the deionized water treated controls (CK), all of the NZVI treatments had significantly larger average lengths. Further investigations with transmission electron microscopy (TEM) and thermogravimetric analysis (TGA) suggested that NZVI particles may penetrate the peanut seed coats to increase the water uptake to stimulate seed germination. The growth experiments showed that although NZVI at a relatively high concentration (320μmol/L) showed phytotoxicity to the peanut plants, the lower concentrations of NZVI particles stimulated the growth and root development of the plants. At certain concentrations (e.g., 40 and 80 μmol/L), the NZVI treated samples were even better than the ethylenediaminetetraacetate-iron (EDTA-Fe) solution, a commonly used iron nutrient solution, in stimulating the plant growth. This positive effect was probably due to the uptake of NZVI by the plants, as indicated in the TEM analyses. Because low concentrations of NZVI particles stimulated both the seedling development and growth of peanut, they might be used to benefit the growth of peanuts in large-scale agricultural settings.

## Introduction

With the rapid development of nanotechnology, iron nanoparticles have been used widely in multiple industrial, commercial, and biomedical applications to benefit society [1,2]. Because of their high reactivity and magnetic property, iron nanoparticles have also been suggested as high-efficiency remediation agents for environmental applications [3–5]. One of the iron nanoparticles, nanosized zero-valent iron (NZVI), is a prominent engineered nanomaterial that has been used extensively in environmental remediation because of the material’s strong ability to degrade organic and other pollutants [4–6]. In the past several years, intentional injections of NZVI suspension into contaminated soil and groundwater systems were promoted in many pilots or full-scale remediation projects worldwide [7,8]. As a result, there are also increased concerns over the environmental impacts of the widespread uses of NZVI in remediation sites [9–11].

A number of studies have explored the environmental impacts, particularly the toxicity of NZVI particles to living organisms [10–12]. Keller tested the toxicity of different types of NZVI to freshwater and marine organisms and reported that the toxicity of NZVI to phytoplankton species and *Daphinia magna* increases with the particle concentrations [11]. El-Temsah and Joner measured the ecotoxicological effects of NZVI coated with carboxymethyl cellulose on two species of earthworms and concluded that doses of more than 500 mg kg^-1^ NZVI are likely to cause acute adverse effects on soil organisms [10]. It has also been reported that relatively high dosages of NZVI can rapidly inactivate *E*. *coli* in aqueous solution or under air saturation by disrupting cell membranes [13]. Diao found, even at low dosages (i.e., 0.1, 1, and 10 mg/L), NZVI can still effectively inactivate soil-based microbes through the combination of several potential mechanisms, including physical coating, membrane damage, and production of reactive oxygen species (ROS) [14]. In another study, however, Sacca investigated the phenotypical and molecular response of bacteria isolated from the NZVI-treated soil to commercial NZVI exposure and found a negligible bacteriostatic effect and the lack of bactericidal effect at low dosages (1–10 mg/L) [15]. Furthermore, Barnes found addition of NZVI particles, even at a very high concentration (100 mg/L), showed no influence on bacterial community structure [16]. Additional research thus is still need to assess the potential ecotoxicology of NZVI in the environment.

Only a few studies have investigated the phytotoxicity of NZVI despite of the fact that exposure to plants is very likely due to the intentional injection into the soils [17–20]. Germination and shoot and root growth tests are often used in these studies to determine the impact of NZVI exposure to different types of plants, such as ryegrass, barley, lettuce, cattail, and poplars [17,19,20]. A recent study by Kim showed that exposure of plants to 500 mg/L NZVI can enhance root elongation because NZVI induces cell wall loosening [18]. Ma found that NZVI at concentrations higher than 200 mg/L showed toxic effects, but it enhanced plant growth at lower concentrations [19]. El-Temsah and Joner reported that only high dosages of NZVI (> 300 mg/L) have an inhibiting effect, while lower concentrations of NZVI can be used without detrimental effects to plants [17]. Similarly, Trujillo-Reyes found that NZVI at low concentrations (< 20 mg/L) did not affect plant physiological parameters and the toxicological effects were produced mainly by the accumulation of iron [20]. Based on previous findings, we concluded that low dosages of NZVI may not only have no toxic effect, but may even have positive effects on plants. Because only a limited amount of investigations have been conducted NZVI phytotoxicity, and most of them were at relatively high NZVI concentration ranges [17–19], the real effects of NZVI exposure to plants at low concentrations are still unclear.

The overarching objective of this work was to determine the effects of low dosage exposure of NZVI on peanut plants. The borohydride reduction method was used to prepare the NZVI particles [21]. To evaluate its biological effects to the plant, NZVI was applied at different concentrations (0–320 μmol/L) to peanut seeds. It is our central hypothesis that low-concentration NZVI may have positive effects on the seed germination and growth of peanut. Laboratory experiments were conducted to test this hypothesis, and the specific objectives were to determine the effects of low-concentration NZVI on: 1) the germination and development of peanut seeds, 2) the growth of peanut seedlings, and 3) the root development of peanut seedlings.

## Materials and Methods

### NZVI

The NZVI particles were prepared using the borohydride reduction method according to the procedures of Ponder [22]. Deionized (DI) water was first purged with N_2_ for 30 min to remove the dissolved oxygen. Under N_2_ atmosphere, 10 g of analytical grade FeSO_4_·7H_2_O was dissolved in 100 mL of 30% technical grade ethanol and 70% DI water (v/v). Polyvinylpyrrolidone (PVP-K30, 5%) was added to the solution as dispersion agent. The pH of the solution was adjusted to about 6.8 with 3.8 M NaOH. KBH_4_ powder (1.8 g) was added incrementally and the mixture was stirred using a homogenizer (Ultra Turrax IKA T25, IKA Instrument Equipment Co., LTD, Germany) for 20 min, followed by filtration using 0.2 μm membrane filter (Millipore). The particles were washed with DI water, rinsed with acetone to remove the excess of NaBH_4_ and water, and vacuum dried (DZG-6050SA, Shanghai SumSung Laboratory Instrument Co., LTD, China). All the chemical reagents were obtained from Tianjin Kemiou Chemical reagent Co., Ltd.

The resulting NZVI particles were suspended in DI water and dispersed with the aid of ultrasonication for 30 min (TH-50, Jining Tianhua ultrasonic instrument Co., LTD, China). For each experiment, a fresh NZVI suspension of 320 μmol-Fe/L (17.92 mg/L) was first prepared and then diluted into a series of low concentration NZVI suspensions (10, 20, 40, 80, 160, 320 μmol/L, or 0.56, 1.12, 2.24, 4.48, 8.96, 17.92 mg/L) for the seed germination and plant growth tests. The treatments of the NZVI suspensions were designated by their concentrations such as NZVI-10, NZVI-20, and NZVI-40 etc.

Morphology of the NZVI particles was examined with a transmission electron microscopy (TEM) at 120 kV to confirm their size distributions. X-ray diffractometer (XRD) analyses were conducted to determine the crystal structures of the NZVI particles. The NZVI material was characterized by powder X-ray diffraction (XRD) using a Rigaku diffractometer and monochromatized Cu KR radiation (generator tension = 40 kV, current = 40 mA). Diffractograms were recorded from 5 to 85° (2θ) with a step size of 0.02° and a count time of 5 s per step.

### Germination of peanut seeds

Preselected peanut (*Arachis hypogaea*) seeds were obtained from Shangdong Peanut Research Institute (Qingdao, China). The seeds were placed on cotton-cushioned petri dishes with 20 mL NZVI of different concentrations (i.e., NZVI-10, NZVI-20, NZVI-40, NZVI-80, NZVI-160, or NZVI-320) and DI water as control (CK). For comparison purpose, 20 mL ethylenediaminetetraacetate-iron (EDTA-Fe) solution of 40 μmol/L, a common used plant iron nutrient solution [23], was also included in the testes. The dishes with treated seeds were covered and incubated for 5 days at 25°C in the dark. Four replicates per treatment were used in the tests and five peanut seeds were used in each replicate. Average lengths of the shoots and roots were determined at the end of the experiment after the 5 days of incubation. In addition, we chose seeds treated with 40 μmol/L EDTA-Fe and 40μmol/L NZVI after 2 days of incubation for thermogravimetric analysis (TGA Q500, TA Instruments-Waters LLC). The seeds were cut into small pieces and only 5 mg of the samples were used in the analysis. TGA was carried out in air at a heating rate of 10°C/min. The level of moistures (%) in the seeds was determined by the mass loss at a temperature range of 0–250°C.

### Growth experiments

Quartz sand (0.2–0.26 mm, Tianjin Kemiou Chemical reagent Co., Ltd, China) was used as the growth medium. Before the tests, it was cleaned sequentially by tap water, 20% hydrochloric acid (v:v), and DI water to remove metal oxides and other impurities until the pH of the sand was 7.0, and then dried at 65°C for 48h. Peanut seeds of similar sizes were germinated and grown in the acid-cleaned sand media for 18 days in a growth chamber (GXZ-SMART, Ningbo Jiangnan Instrument Co., China) at 25°C. The growth tests had eight treatments with different concentrations of NZVI (CK, NZVI-10, NZVI-20, NZVI-40, NZVI-80, NZVI-160, NZVI-320, and EDTA-Fe) in modified Hoagland nutrient solutions (no iron nutrient) and only DI water was added to the nutrient solution in the CK treatment [24]. For each test, plastic tubing was placed in the container to replace nutrient solution. 110 g of the sand was first packed into a 100 ml plastic container with one peanut seed placed on the top layer. Another 40 g of sand were then added to the container to cover the seed. To start the growth experiments, 40 mL of the modified Hoagland nutrient solution was added into the container. After that, 5 mL of the modified Hoagland nutrient solution were added into the sand every three days. The plants were grown for 18 days and were removed from the sand.

The roots were carefully washed with DI water and dried with paper tissues before the length and weight measurements and root scan analyses. Three peanut plant samples of each treatment were chosen and washed with DI water. Before the root scan, samples were ultrasonicated in DI water for 2 min (TH-50, Jining Tianhua ultrasonic instrument Co., LTD, China) in order to remove quartz sand from the roots. A Winrhizo root analysis system with a root scanner (PERFECTION V700 PHOTO, Epson co., LTD, Japan) was then used to measure all the samples. For statistical purposes, each treatment of the growth study was replicated at least 4 times.

### TEM analyses

Seed and seedling samples from the germination testes and root samples from the growth experiments were collected for the TEM analyses (JEM-1200EX, JEOL Ltd., Japan). Only the seed and plant tissues of CK and NZVI-40 were analyzed. For seed coat, the inside was analyzed; while the tips were analyzed for roots. The samples were first cut into < 1 mm^3^ pieces using a razor blade and were then rinsed with 0.05 M PIPES buffer solution containing 1.5 mM CaCl_2_ and 1.5 mM MgCl_2_. Finally, the samples were soaked in a fixing solution containing 2% paraformaldehyde, 2.5% glutaraldehyde, 1.5 mM CaCl_2_, 1.5 mM MgCl_2_ and 0.05 M PIPES buffer, and stored at 4°C overnight. After fixing, the samples were dehydrated in an ascending ethanol series from 10 to 70% ethanol, in 10% increments for 20 min for each solution. The samples were permeated with Spurr resin and epoxy propane in a ratio of 1:1 for 1 hour, then with Spurr resin only for 2 h. Using small PCR pipe or EP for mold, the samples were put in resin for embedding at 70°C for 24 h. Cured resin blocks were trimmed, thin sectioned and collected on formvar copper slot grids. Sections were examined with a TEM with working voltage of 80 KV.

### Statistical analyses

All results and standard errors were calculated from four replicates. Differences between the NZVI treatments and CK and EDTA-Fe were tested using one-way analysis of variance (ANOVA) and mean separation tests (Duncan’s multiple range test and least significant difference test (LSD)) were performed using Statistical Analysis System (SAS) package version 9.2 (2010, SAS Institute, Inc., Cary, NC).

## Results and Discussion

### NZVI Characterization

**Fig 1:**
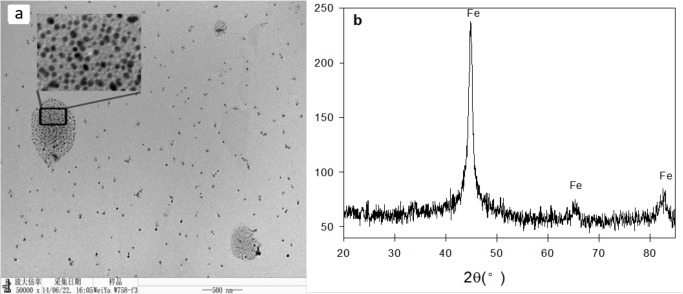
TEM (a) and XRD (b) analyses of NZVI.

TEM and XRD analyses were performed to characterize the synthesized NZVI particles. The TEM images showed that individual particles ranging in size from 20 nm to 80 nm using Image J 1.44 (Fig 1A), confirming the success of the borohydride reduction method. The XRD diffraction peaks at 44.9°, 65.0°, and 85.0° (2θ) could be assigned to zero-valent iron particles [25,26].

### Effects of NZVI on germination

**Fig 2:**
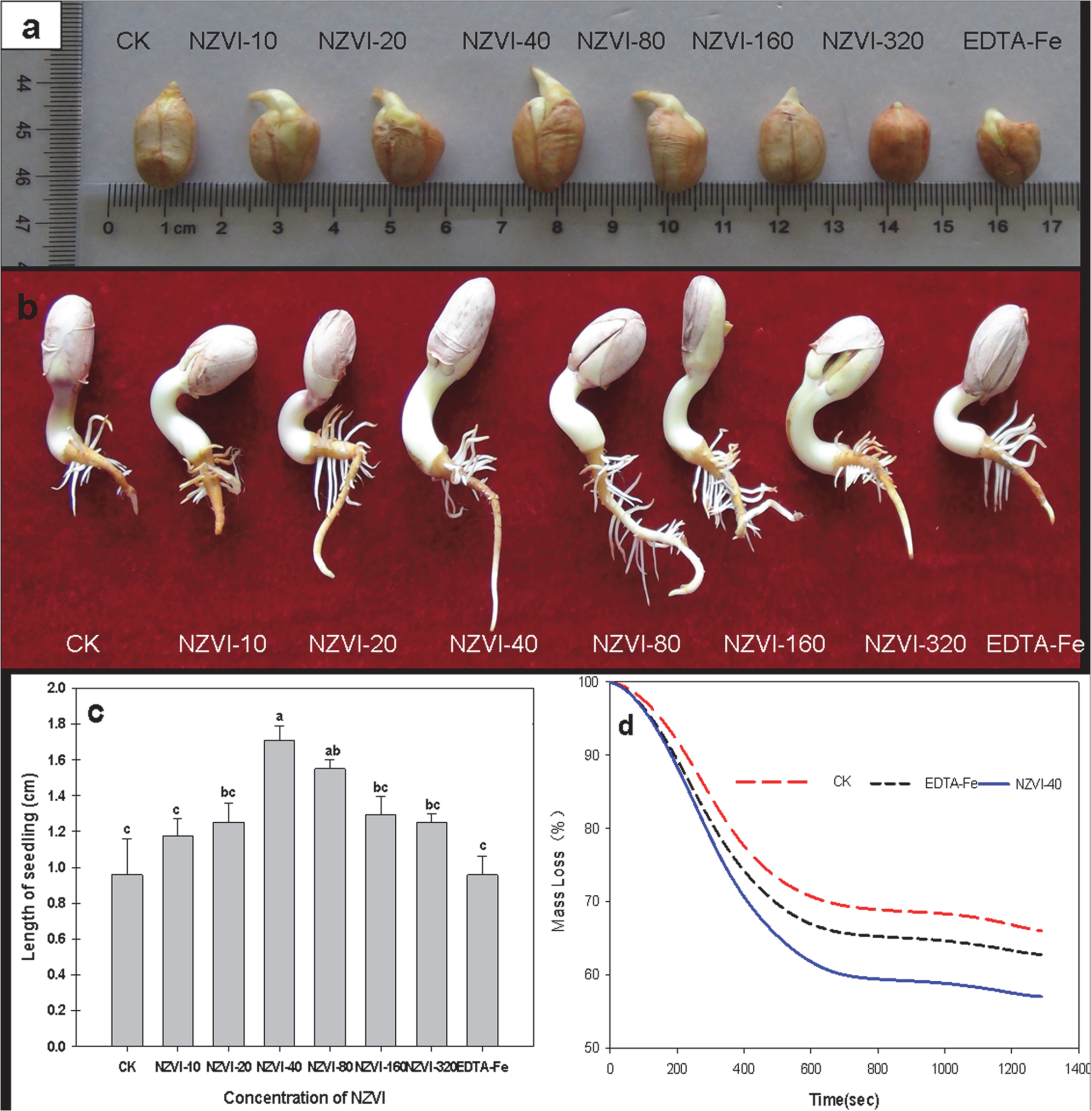
Effects of NZVI treatments on peanut seed germination. a) reprehensive photograph of seeds after 2 days, b) reprehensive photograph of seedlings after 4 days, c) length of the seedlings after 4 days, and d) TGA analyses of selected seeds after 2 days. Different letters show significant differences (p⩽0.05) and bars represent the standard error of treatments (n = 4). NZVI was indicated by dark arrow.

Because selected peanut seeds were used in the experiment, all the seeds germinated regardless of the treatment methods. After two days of incubation, all the treatments showed the signs of the development of embryonic roots (Fig 2A). At the end of the experiment, seedlings with embryonic root and shoot systems were observed for all the tested seeds (Fig 2B), suggesting the NZVI may not have strong toxic effects on seed germination. In a previous study, El-Temsah and Joner also reported that NZVI has no significant effect on seed germination rates of several plant species at concentrations lower than 250 mg/L [17]. The highest NZVI concentration used in this study was 320 μmol/L (i.e., 8.3 mg/L), far below that threshold. As a result, none of the NZVI treatments showed any effects on the germination rates of the peanut seeds in this work.

The NZVI exposure, however, showed notable effects on the development of the roots (seedlings). Measurements of the seedling lengths indicated the NZVI treatment promoted root growth (Fig 2C). The average seedling lengths of the NZVI treated peanut seeds were significantly larger than that of the control groups, not only the CK group but also the EDTA-Fe. Among all the groups, the NZVI-40 showed the longest seedling (1.7 cm), which was almost twice of that of the CK (0.9 cm) and EDTA-Fe (0.9 cm). These results suggested that low concentrations of NZVI promoted the embryonic growth of the peanut seeds. Previous studies have shown that engineered nanoparticles, such as carbon nanotubes, can also promote seed germination and growth because the particles can penetrate the plant seed coat to support water uptake [27–29]. This mechanism could also be applied to explain the findings of this work. Several studies have suggested that NZVI particles can generate reactive oxygen species (ROS), such as OH radical [4,5,18]. When plant seeds are treated with NZVI particles, the induced radicals thus may thin or open plant seed coats. For example, Kim reported that NZVI-mediated OH radical can induce cell wall loosening to enhance *Arabidopsis thaliana* root elongation by 150–200% over that of the control (i.e. without NZVI under same conditions) [18]. Based these findings, it seemed that the NZVI particles could also penetrate the peanut seed coats to increase the water uptake and thus promote seed germination activity by increasing the yield (seedling development).

This hypothesis was confirmed by the results from the TGA analyses of seed moisture after two days of incubation (Fig 2D), which showed that the NZVI-40 treated peanut seeds contained much more water than that of the CK or EDTA-Fe group. Under the experimental conditions, the mass loss in the TGA curve was corresponding to the moisture content of the seeds. Thus, the moisture levels of the CK, EDTA-Fe, and NZVI samples were 30%, 35% and 45%, respectively (Fig 2D). This result indicated that NZVI could significantly enhance the water uptake inside tomato seeds. It could be based on the assumption that NZVI are able to penetrate seed coat while supporting and allowing water uptake inside the seed.

**Fig 3:**
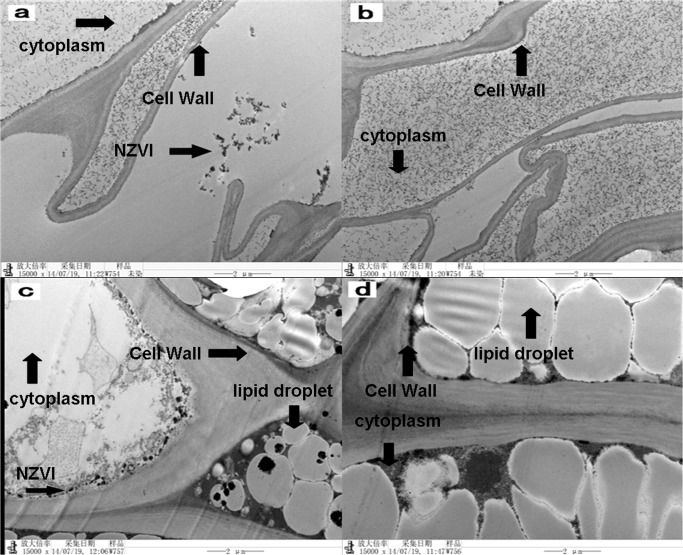
TEM analyses of peanut seeds after 4 days. a) seed coat of NZVI-40, b) seed coat of CK, c) peanut of NZVI-40, and d) peanut of CK. The dark arrows show small clusters of NZVI particles. NZVI was indicated by dark arrow.

TEM images of seed coats (inside) and peanut cotyledon treated by NZVI showed that NZVI is inside the cell wall (Fig 3). This phenomenon confirmed that the NZVI can loosen or open the peanut seed coat. Small clusters of particles (indicated by dark arrow in Fig 3A) were found within the seed coat samples treated with NZVI-40. The black particles might be NZVI particles, because they were not found in the CK samples (Fig 3B). In addition, TEM images of plant tissue samples treated with the NZVI-40 (Fig 3C) and CK (Fig 3D) also clearly demonstrated the presence of clusters of the nanoparticles (indicated by dark arrow in Fig 3C) in treated tissues. These results indicated that NZVI particles can penetrate plant seed coats and might be utilized by the plants. In a recent study, Trujillo-Reyes has also reported the uptake and accumulation of NZVI by lettuce plants.

### Effects of NZVI on growth

**Fig 4:**
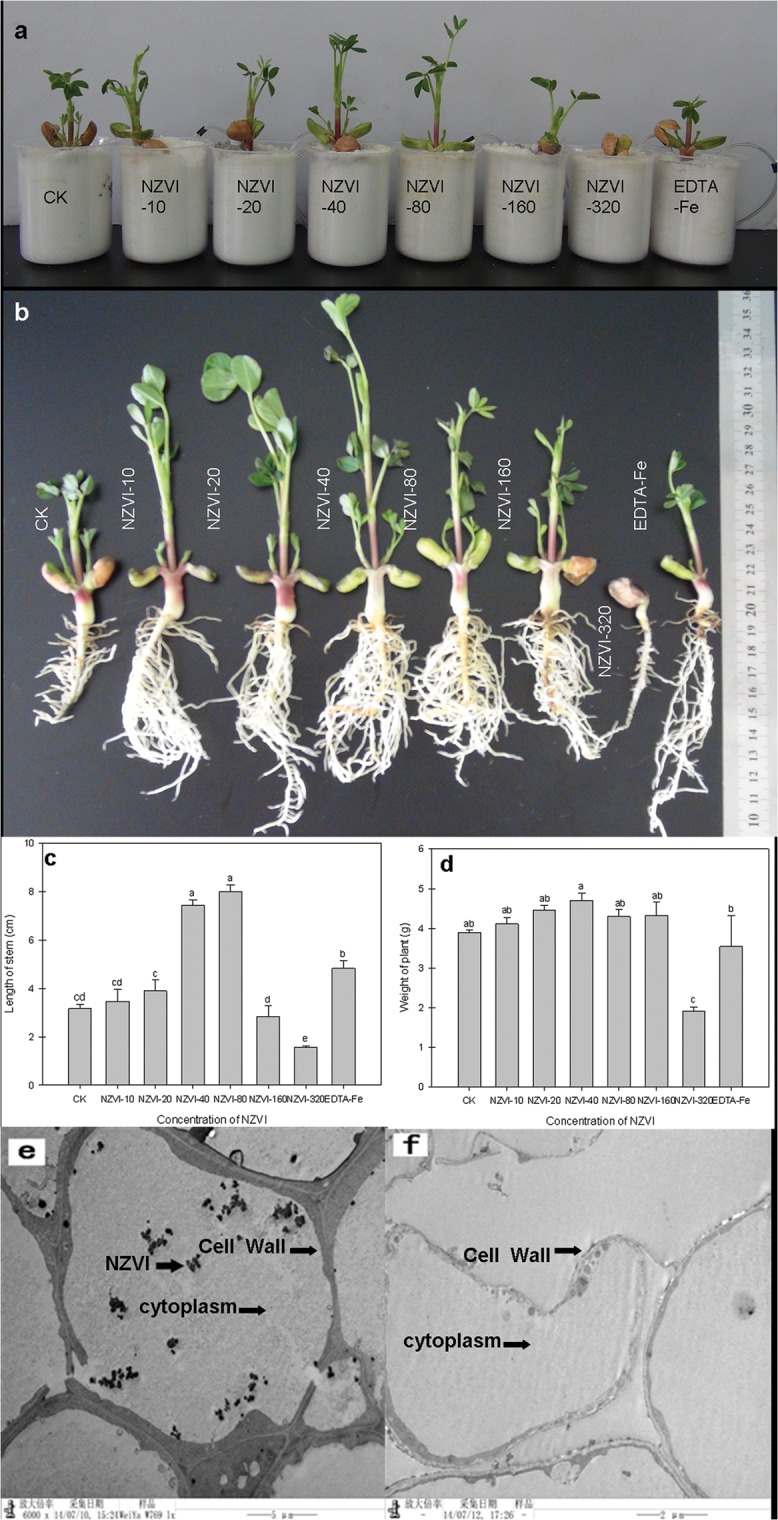
Effects of NZVI treatments on peanut plant growth. a) photograph of peanut plants in containers after 13 days, b) photograph of peanut plants after 18 days, c) length of the stems of the peanut plants, d) weight of the peanut plants, e) TEM image of root tissue of NZVI-40, and f) TEM image of root tissue of CK. Different letters show significant differences (p⩽0.05) and bars represent the standard error of treatments (n = 4). NZVI was indicated by dark arrow.

After 18 days, most of the peanut plants grew very well except the ones treated with NZVI-320. These plants showed no obvious sign of shoot development (Fig 4A and 4B). This result indicated that high dosage of NZVI may have negative effects on the growth of the plants. Measurement of the plant stem length (Fig 4C) and total biomass (Fig 4D) further confirmed the inhibition effects of NZVI-320 to the growth of peanuts. Both the average length and biomass weight of NZVI-320 treated plants were significantly lower (*p* < 0.05) than that of the control group. Although phytotoxicity of NZVI has been observed for various types of plants such as cattail, poplar, and lettuce [19,20], it also occurs at very high concentrations (e.g., > 300 mg/L). Results obtained from the growth experiments, however, suggested that even at low concentration (i.e., 320 μmol/L or 17.92 mg/L), NZVI may still have phytotoxicity to peanut plants. Therefore, special attention should be paid to monitoring NZVI particles when they are being intentionally injected into soil and groundwater systems.

On the other hand, the treatments with lower NZVI concentrations (i.e., 10–160 μmol/L) showed no signs of plant growth inhibition and some treatments even stimulated growth (Fig 4A and 4B). The average stem lengths of the plants treated with NZVI-40 and NZVI-80 were not only twice of that of the CK group, but also significantly larger (p < 0.05) than the samples treated with EDTA-Fe, the 40 μmol/L iron nutrient solution (Fig 4C). In addition, average biomass weights of the plants treated with lower NZVI dosages (i.e., 10–160 μmol/L) were generally higher than that of the CK and the EDTA-Fe samples. The NZVI-40 samples had the highest average weight, which was significantly (p < 0.05) larger than that of both CK and EDTA-Fe samples. Based on these results, it can be concluded that low concentrations of NZVI may be used to simulate the growth of peanut plants under certain conditions. The stimulation effects of the NZVI to the peanut plant probably could be attributed to the uptake of NZVI particles by the plants [20]. At relatively low concentrations, the NZVI particles in the plants may be used to stimulate growth. The TEM image analyses of the peanut plant tissues showed small clusters of particles in root tips (Fig 4E). The particles were present in cytoplasm and inside the cell wall. Because the particle clusters were not found in the CK samples (Fig 4F), they could be the NZVI aggregates. These images thus confirmed the uptake of NZVI by the peanut plants.

**Fig 5:**
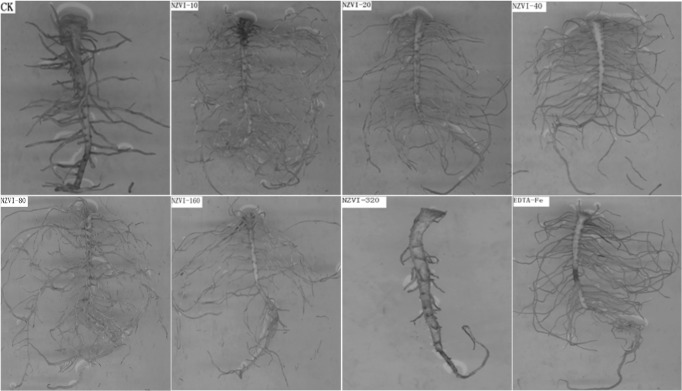
Images of roots of peanuts with different NZVI treatments.

Plant root images (Fig 5) confirmed the phytotoxicity of the NZVI-320 to the peanut plants, which is consistent with the literature that high concentration of NZVI may have toxicological effects to plants due to the potential uptake and accumulation of the nanoparticles [20]. In comparison with the other samples, the root of NZVI-320 treated sample was not well-developed. The root scanning analyses showed that lower concentration (i.e., 10–160 μmol/L) NZVI particles promote the growth and development of peanut root systems (Table 1). As shown in the table, the NZVI-10 to NZVI-160 treated samples all had much longer roots, larger projected area, larger surface, larger volume, more tips, more forks, and more crossings than the CK treated samples. Those values of the NZVI-40 and NZVI-80 treated samples were even higher than that of the EDTA-Fe treated samples. The results from root analyses also suggested that low concentrations of NZVI can be used to simulate the root development of peanut plants under certain conditions.

**Table 1:** Effects of NZVI treatments on the root development of peanut plant.

Treatments	Sum of Length(cm)	Projected Area(cm^2^)	Surface Area (cm^2^)	Average Diameter(mm)	Root Length per Volume (cm/m^3^)	Root Volume(cm^3^)	Number of Tips	Number of Forks	Number of Crossings
CK	54.4±0.79 d	5.3±0.11d	16.8±0.33d	0.9±0.02a	54.4±0.79d	0.4±0.01c	97.0±1.53d	235.0±2.73d	26.0±3.61c
NZVI-10	172.1±9.42 bc	17.6±1.05bc	55.4±3.29bc	0.9±0.01a	180.3±9.42abc	1.3±0.09b	498.0±44.55bc	1244.0±43.54bc	150.0±41.51b
NZVI-20	180.3±22.68 abc	19±3.23abc	59.7±10.14abc	1.0±0.13a	172.1±28.82abc	1.6±0.28ab	492.0±155.36bc	1155.0±320.12c	186.0±20.99ab
NZVI-40	217.2±3.59 ab	22.2±0.78ab	70.1±2.44ab	1.1±0.02a	217.2±3.59ab	1.8±0.10ab	782.0±44.79a	1661.0±92.39ab	201.0±21.07ab
NZVI-80	226.5±28.82 a	24.0±1.54a	75.6±4.85a	1.0±0.03a	226.6±22.68a	2.1±0.34a	745.0±120.25ab	1786.0±187.65a	229.0±35.10a
NZVI-160	167.0±5.14 c	16.1±0.61c	50.7±1.90c	0.9±0.01a	167.0±5.13c	1.2±0.06b	491.0±49.96bc	1029.0±59.50c	146.0±11.85b
NZVI-320	11.9±4.26 d	1.0±0.31d	3.4±0.96d	0.9±0.24a	11.9±4.26d	0.1±0.03c	18.0±9.53d	53.0±23.10d	5.0±1.45c
EDTA-Fe	182.8±20.16 abc	17.7±3.15bc	55.6±9.9abc	0.9±0.08a	182.8±20.16abc	1.4±0.33b	465.0±118.08c	1047.0±222.95c	144.0±37.53d

Different letters in the same column show significant differences (p⩽0.05)and n = 4.

## Conclusions

As the first of its kind, this work showed that low concentrations of NZVI stimulated not only seed germination but also the growth of peanut plants. Because iron deficiency occurs in a variety of soils, it causes serious problems to various types crops including peanuts [30–32]. Low concentrations of iron nutrients, such as EDTA-Fe, thus are often used as a fertilizer to enhance the yield [31,33]. Findings from this work suggested that low concentrations of NZVI may be used to stimulate seed germination and the growth of peanut plants.

## Supporting Information

S1 Table The raw data of the length of seedling, TGA, length of stem, weight of the plants and root scan analyses. (XLS)
